# Hair Cell Regeneration after ATOH1 Gene Therapy in the Cochlea of Profoundly Deaf Adult Guinea Pigs

**DOI:** 10.1371/journal.pone.0102077

**Published:** 2014-07-18

**Authors:** Patrick J. Atkinson, Andrew K. Wise, Brianna O. Flynn, Bryony A. Nayagam, Rachael T. Richardson

**Affiliations:** 1 Bionics Institute, East Melbourne, Victoria, Australia; 2 Department of Otolaryngology, University of Melbourne, East Melbourne, Victoria, Australia; 3 Department of Medical Bionics, University of Melbourne, East Melbourne, Victoria, Australia; 4 Department of Audiology and Speech Pathology, University of Melbourne, Parkville, Victoria, Australia; Texas A&M University, United States of America

## Abstract

The degeneration of hair cells in the mammalian cochlea results in permanent sensorineural hearing loss. This study aimed to promote the regeneration of sensory hair cells in the mature cochlea and their reconnection with auditory neurons through the introduction of ATOH1, a transcription factor known to be necessary for hair cell development, and the introduction of neurotrophic factors. Adenoviral vectors containing ATOH1 alone, or with neurotrophin-3 and brain derived neurotrophic factor were injected into the lower basal scala media of guinea pig cochleae four days post ototoxic deafening. Guinea pigs treated with ATOH1 gene therapy, alone, had a significantly greater number of cells expressing hair cell markers compared to the contralateral non-treated cochlea when examined 3 weeks post-treatment. This increase, however, did not result in a commensurate improvement in hearing thresholds, nor was there an increase in synaptic ribbons, as measured by CtBP2 puncta after ATOH1 treatment alone, or when combined with neurotrophins. However, hair cell formation and synaptogenesis after co-treatment with ATOH1 and neurotrophic factors remain inconclusive as viral transduction was reduced due to the halving of viral titres when the samples were combined. Collectively, these data suggest that, whilst ATOH1 alone can drive non-sensory cells towards an immature sensory hair cell phenotype in the mature cochlea, this does not result in functional improvements after aminoglycoside-induced deafness.

## Introduction

Hearing loss is a major health concern which affects over 5% of the world's population. This equates to approximately 360 million people suffering from a disabling hearing impairment (World Health Organisation, 2013), a number that is expected to grow with an ageing population. Sensorineural hearing loss (SNHL), the most common cause of hearing loss, can occur as a result of a congenital defect or be acquired through exposure to excessive noise, exposure to certain classes of antibiotics, infections or ageing. The loss of hearing in many of these cases is permanent due to the irreversible degeneration of the sensory hair cells (HCs) in the cochlea [1]. Currently, the only clinical treatment for a severe-to-profound SNHL (characterised by a hearing threshold of 70 dB or above) is a cochlear implant, which bypasses the damaged or lost HCs and electrically stimulates the remaining auditory neurons. Despite their success, however, there is variable patient performance with a cochlear implant, especially in relation to speech perception in noisy environments and for music appreciation, where performance can be markedly decreased [2], [3]. As such, in the last decade there has been a strong research focus on alternative treatments for SNHL, in particular, the use of novel techniques to restore the degenerated elements of the cochlea.

Hair cell regeneration is thought to be the panacea for restoring function to the cochlea after SNHL, however, this is not without challenges. For example, it is known from developmental studies that the initial formation, patterning and correct connection of HCs to auditory neurons requires a complex cascade of molecular signaling with precise timing [4]. One of these molecular signals is the expression of the basic helix-loop-helix transcription factor ATOH1, a factor which has been found to be necessary for HC development and is thought to be the earliest determinant of HC fate [5], [6]. Indeed, ATOH1 null mice lack both cochlear HCs and the supporting cells that comprise the sensory epithelium known as the organ of Corti (OC) [7]. Moreover, the overexpression of ATOH1 has been shown to result in ectopic and supernumerary HCs, which is thought to occur through the direct transdifferentiation of non-sensory supporting cells in the OC towards a HC fate [7]–[9]. Experimental manipulations reintroducing ATOH1 into the deaf cochlea has also highlighted the role of this transcription factor in HC development. An initial study in the short-term (four day) ototoxically deafened guinea pig (GP), demonstrated both a greater number of HCs in the viral-mediated ATOH1-treated cochlea (as noted by the expression of a known HC marker, myosinVIIa) and also a significant improvement in hearing thresholds (as measured by auditory brainstem responses; ABRs) [10]. These results in the mature GP provided evidence that it is possible to manipulate non-sensory cells to generate sensory HCs, which subsequently leads to improved auditory function. These findings were further bolstered by a gain-of-function study, which demonstrated that *in utero* gene transfer of ATOH1 not only forced the production of supernumerary and ectopic HCs, but also that these additional HCs were functional [8]. Whilst, these results were very promising, recent studies have underscored the complex nature of HC regeneration. Indeed, the ability to regenerate HCs, particularly within the mature cochlea, has been variable [10]–[13]. Further to this, a recent study demonstrated that ATOH1-induced HC regeneration after HC loss is possible in neonatal and juvenile mice but not in adult mice [13]. Moreover, little is known about the ability of these newly generated HCs to form synaptic connections with remaining auditory neurons, especially within the mature cochlea, which is critical for the effectiveness of this strategy. A recent study, however, has demonstrated that the presence of NTs significantly enhanced synaptogenesis between HCs and the regrowing peripheral fibres after deafferentation [14].

Experiments carried out in this study examined the ability of ATOH1 gene therapy alone or in combination with NT-gene therapy, to induce transdifferentiation of non-sensory cells towards a HC phenotype in the mature deaf cochlea and promote the regrowth of peripheral fibres towards the new HCs. Transduced cells were further characterised, with a particular focus on the ability of these cells to generate complex ribbon synaptic proteins, attract auditory neuron peripheral fibres and improve hearing.

## Materials and Methods

### Ethics Statement

National Health and Medical Research Council (NHMRC) and Nation Institutes of Health (NIH) Guidelines for the Care and Use of Laboratory Animals were observed. The Animal Research Ethics Committee of the Royal Victorian Eye and Ear Hospital approved the care and use of the animals in this study (ethics #09/180AB).

### Adenoviral vectors

Adenoviral vectors were generated as previously described [15]. Briefly, replication deficient adenovirus type 5 was genetically modified to expression green fluorescent protein (GFP) in concert with mouse brain derived neurotrophic factor (BDNF), mouse neurotrophin-3 (NT-3) or mouse ATOH1. Adenoviral vectors were diluted 1∶5 in artificial endolymph (120 mmol/l KCL, 2.5 mmol/l NaCl, 0.5 mmol/l MgCl_2_, 028 mmol/l CaCl_2_, 7.6 mmol/l K_2_HPO_4_, 2.7 mmol/l KH_2_PO_4_, pH 7.4) to final concentrations of 3.0×10^10^ OPU/ml (Ad-NT-3), 4.3×10^10^ OPU/ml (Ad-BDNF) and 2.1×10^11^ OPU/ml (Ad-ATOH1). Ad-NT-3 and Ad-BDNF were mixed in a 1∶1 ratio just prior to injection and will hereafter be referred to as Ad-NTs. When required Ad-NTs was mixed in a 1∶1 ratio with Ad-ATOH1 (Ad-ATOH1+Ad-NTs).

### Experimental animals

Male and female adult pigmented Dunkin-Hartley GPs (n = 26, average weight 365±12 g) were used in this study. Each gene therapy group had five animals while the normal and four day deaf groups had three animals (in the normal and four day deaf groups data was used from left and right cochleae as there was no difference in HC number observed). Viral administration was performed with the approval of the Office of the Gene Technology Regulator Australia (Licence #444), and under approval of the Royal Victorian Eye and Ear Hospital Animal Ethics Committee.

### Deafening

The hearing status of each GP was assessed prior to deafening by measuring ABRs to computer-generated click and tone-pip stimuli (1, 2, 8, 16, 24 and 32 kHz) [16]. For inclusion in the study, GPs were required to have normal hearing, which was defined as an auditory brainstem response (ABR) threshold <43 dB peak-equivalent sound pressure level. Animals meeting this criterion were deafened under gaseous anaesthesia via intravenous infusion of 100 mg/kg furosemide (Troy Laboratories, Smithfield, Australia) and subcutaneous injection of 400 mg/kg kanamycin sulphate (Applichem, Taren Point, Australia; [17]). Deafness was confirmed four days post-procedure using ABRs, where animals with threshold shifts of >50 dB were considered profoundly deaf.

### Cochlear injection of viral sample

In order to target the supporting cells of the OC two microliters of Ad-GFP, Ad-NTs, Ad-ATOH1 or Ad-NTs+Ad-ATOH1 were unilaterally injected into the lower basal scala media of the cochlea four days post-deafening as previously described [15]. Briefly, a retroauricular approached was used to expose the bulla. An opening was made in the bulla with a drill and a small cochleostomy was made into the otic capsule of the cochlear basal turn. Perilymph was removed using gentle suction to visualise basilar membrane. A glass recording micropipette was advanced via a stepper motor (Kopf, model 2662) through the basilar membrane until an endocochlear potential was measured, after which the viral preparation was injected using a rate controlled micropump system (WPI), over 5 minutes. This approach was selected to specifically target the OC for gene transfection. The pipette was then retracted and the cochleostomy sealed with connective tissue, the bulla sealed with dental cement and the wound closed in layers with dissolvable sutures.

### Functional assessment and histology

Four days post-deafening and three weeks post-injection GP hearing was assessed with click and tone-pip ABRs as described above. Animals were then euthanised with 1.5 ml pentobarbitone and intracardially perfused with 0.9% (wt/vol) saline containing 0.1% (vol/vol) heparin sodium and 0.025% (wt/vol) sodium nitrite, followed by 10% (vol/vol) neutral buffered formalin. The bullae were removed and the cochleae visualised. Cochleae were decalcified, embedded in optimal cutting temperature compound and sectioned in the pre-modiolar and mid-modiolar planes as previously described [18]. Half of the cochlea was retained for surface preparations as described [15], [19]. The following primary antibodies were used: anti-neurofilament (1∶200, NF; Merck Millipore, Australia), rabbit anti-myosinVIIa (1∶100, Proteus Bioscience), mouse anti-myosinVIIa (1∶100, Developmental Studies Hybridoma Bank, University of Iowa) anti-calbindin (1∶500, Merck Millipore), anti-parvalbumin (1∶200, Sigma), anti-prestin (1∶50, Santa Cruz Biotechnology), anti-oncomodulin (1∶200, Swant), anti-CtBP2 (1∶50, BD Biosciences). AlexaFluor secondary antibodies (1∶200–1∶500, Life Technologies) were used to visualise several antibodies in the same sample and mounted in media with or without DAPI. Sections were examined on a Zeiss Axioplan fluorescence microscope and imaged using Axiovision v4.8 (Carl Zeiss, Germany). Cochlear half-turn surface preparations and pre mid-modiolar sections were viewed on a Zeiss LSM Meta 510 confocal microscope and images captured using Zen software (2009 edition).

## Data Analysis

### Hair cell quantification

Hair cells were analysed in basal turn surface preparations. Cells which expressed MyosinVIIa, had a nucleus, and resided within the organ of Corti were categorised as HCs. These cells were quantified along the entire length of the basilar membrane. The length of the basilar membrane was measured and the total number of HCs was expressed per mm of basilar membrane.

### Quantification of presynaptic ribbons

Inner hair cell synaptic ribbon quantification was conducted using basal turn surface preparations. Optical sections in the z-axis were imaged and recorded at 0.6 µm intervals for high resolution. Pre-synaptic ribbon synapses were visualised as CtBP2 immunoreactive puncta. The number of synaptic ribbons observed in each inner hair cell (IHC) was quantified by counting CtBP2 puncta within an IHC. The percentage of IHCs with CtBP2 puncta was also quantified. For all Ad treatment groups, every HC was imaged for quantification.

### Quantification of peripheral fibre regrowth towards transduced cells

The growth of auditory neuron peripheral fibres towards transduced cells in the scala media was analysed from confocal images that contained GFP-positive cells, as previously described [18]. Briefly, confocal images were initially split into their individual channels using ZEN software. These images were converted to 32-bit and thresholded using the “moments” algorithm in ImageJ. This was done for each image within the stack. The GFP-positive channel image was opened in Gimp software (GNU image manipulation software) and a 10 pixel border was drawn around the GFP-positive cell. This border was then copied and pasted on the NF-positive image, such that only those NF-positive fibres that fell within this area were visible. The pixel density of these fibres was then measured using ImageJ software. Four consecutive Z-planes with the highest pixel density were averaged to give a final result. Only GFP-expressing cells within the OC region and distal to the IHCs were used for analysis to avoid including fibres that are normally observed in the inner spiral bundle, as such, not all animals could be included in this analysis (n = 5 GPs for Ad-GFP, n = 4 GPs for Ad-NTs). Measurements from Ad-GFP injected GPs and Ad-NTs injected GPs were compared using a student's t-test.

### Auditory neuron density

Spiral ganglion neuron density was analysed blindly from three non-consecutive (greater than 72 µm apart) mid-modiolar sections from each cochlea as outlined previously [18]. Briefly, density was calculated by counting co-labelled NF and DAPI-positive auditory neuron cell bodies within Rosenthal's canal, and then dividing the number of auditory neurons by the area of Rosenthal's canal using ImageJ software. Lower and upper basal, middle and apical turn densities were averaged to calculate the overall density for each cochlear region. Statistical analyses of auditory neuron density data were performed using a paired student's t-test and presented as mean ± SEM.

## Results

### Hair cell regeneration with ATOH1 gene therapy

**Figure 1:**
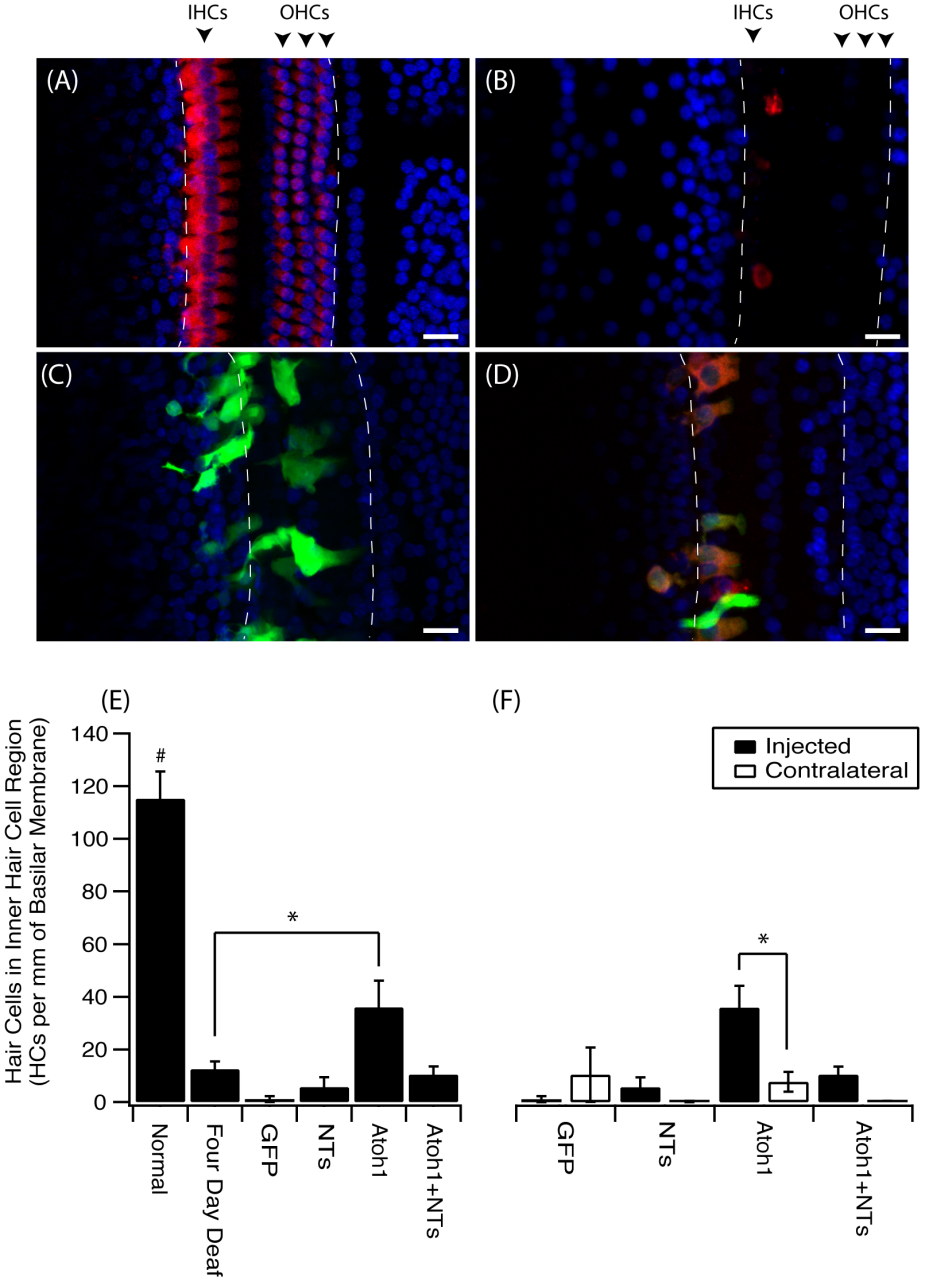
An increase in hair cells in the inner hair cell region after ATOH1 gene therapy. Example photomicrographs of surface preparations of the basal turn of the cochlea from (A) normal, (B) four day deaf, (C) Ad-GFP treated, (D) Ad-ATOH1 treated animals (red  =  myosinVIIa, green  =  GFP, blue  =  DAPI, dashed lines mark the sensory region, scale bar  = 20 µm). (E) When quantified there was a significant loss of IHCs after four days of deafness compared to normal hearing animals. Three weeks post ATOH1-gene therapy there were significantly more myosinVIIa positive cells in the IHC region compared with the four day deaf group (*p<0.05, ANOVA). This number however remained below that observed in normal cochleae which had a greater number of IHCs compared to any other treatment group (#p<0.05, ANOVA). (F) A comparison of treated (injected) versus non-treated (contralateral) cochleae showed ATOH1-injected cochleae to have a significantly greater number of IHCs relative to the contralateral control cochleae (§p<0.05 paired t-test).

In normal hearing cochleae there was a well-defined single row of myosinVIIa-positive IHCs displaying stereotypical pear-shaped morphology and three rows of myosinVIIa-positive outer hair cells (OHCs), along the entire length of the surface preparation (Figure 1A). Animals that were sacrificed four days post-deafening showed a complete bilateral loss of OHCs and a near-complete loss of IHCs in the basal turn. The few remaining IHCs in these animals had atypical spherical morphology (Figure 1B). Deafened cochleae examined three weeks after Ad-GFP (Figure 1C) or Ad-NTs treatment also had very few HCs compared normal hearing animals, despite significant regions of GFP expression. In contrast, cochleae treated with Ad-ATOH1 displayed myosinVIIa and GFP co-positive HCs in the lower basal turn. These HCs had a varied morphology with some displaying a typical pear-shaped morphology and others with a more spherical morphology (Figure 1D). The HCs observed in ATOH1 treated cochleae were generally located proximal to the IHC region with some ectopic expression observed near the inner spiral bundle and in the outer sensory region, proximal to the location of the OHCs.

There were no significant differences detected between the number of HCs observed after four days of deafness alone compared to Ad-GFP or Ad-NTs treated animals (Figure 1E). The Ad-mediated introduction of ATOH1, in contrast, resulted in significantly more myosinVIIa-positive cells compared to the four day deaf cohort (Mann-Whitney test, p<0.05; Figure 1E) and their contralateral non-treated cochleae (Student t-test, p<0.05; Figure 1F). The overall numbers of HCs in ATOH1 treated cochleae was still significantly lower than those observed in normal cochleae. The combined treatment of ATOH1 and NTs did not result in an increase in HCs compared to the four day deaf group. However, the ATOH1 and NT viral titres in this group were halved, limiting the inferences that can be made for this group.

### Characterisation of ATOH1 transduced cells

#### Immunohistochemical characterisation

**Figure 2:**
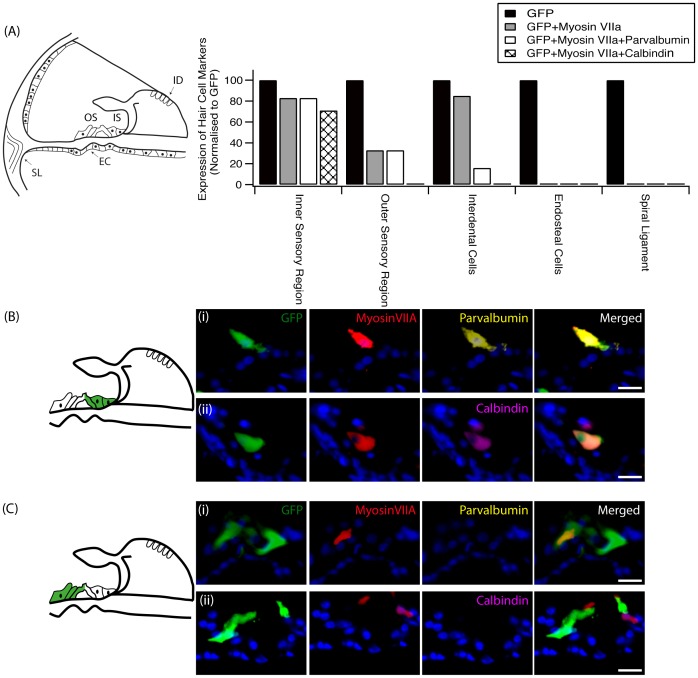
HC marker expression following ATOH1 treatment. (A) The transduction of cells with ATOH1 resulted in cells expressing HC markers to varying degrees depending on their location (EC: endosteal cells, ID: interdental cells, IS: inner sensory region, OS: outer sensory region, SL: spiral ligament). Transduced cells (green) in the inner sensory region (B) had the highest percentage of cells that expressed the HC markers myosinVIIa (red), parvalbumin (yellow) and calbindin (magenta). A high proportion of myosinVIIa-positive transduced cells in this region co-labelled with (i) parvalbumin or (ii) calbindin. Fewer cells in the outer sensory region (C) expressed HC markers. ATOH1-transduced cells within the spiral ligament or endosteal cell region did not express any HC markers. Scale bar  = 20 µm.

To more thoroughly characterise cells that had been transduced by ATOH1, mid-modiolar sections were double-labelled with myosinVIIa and either parvalbumin, calbindin, oncomodulin or prestin (Figure 2). The location of transduced cells was further divided into five regions consisting of the inner sensory region (proximal to the location of the IHCs), the outer sensory region (proximal to the location of the OHCs), the interdental cell region of the spiral limbus, the spiral ligament region and finally the endosteal cell region. In ATOH1-transduced cells in the inner sensory region, which had the most transduced cells, parvalbumin was found to co-label with myosinVIIa 83% of the time and calbindin co-labelled with myosinVIIa 71% of the time. In the outer sensory region this percentage dropped to 33% of transduced cells for myosinVIIa and parvalbumin co-labelling, and cells were no longer positive for calbindin. The myosinVIIa, parvalbumin and calbindin HC markers were not expressed in any ATOH1-transduced cell in the endosteal and spiral ligament regions. Moreover, regardless of location, ATOH1-transduced cells failed to express the mature HC markers oncomodulin or prestin (data not shown). When transduced with Ad-GFP only, no co-localisation with any HC marker was observed.

**Figure 3:**
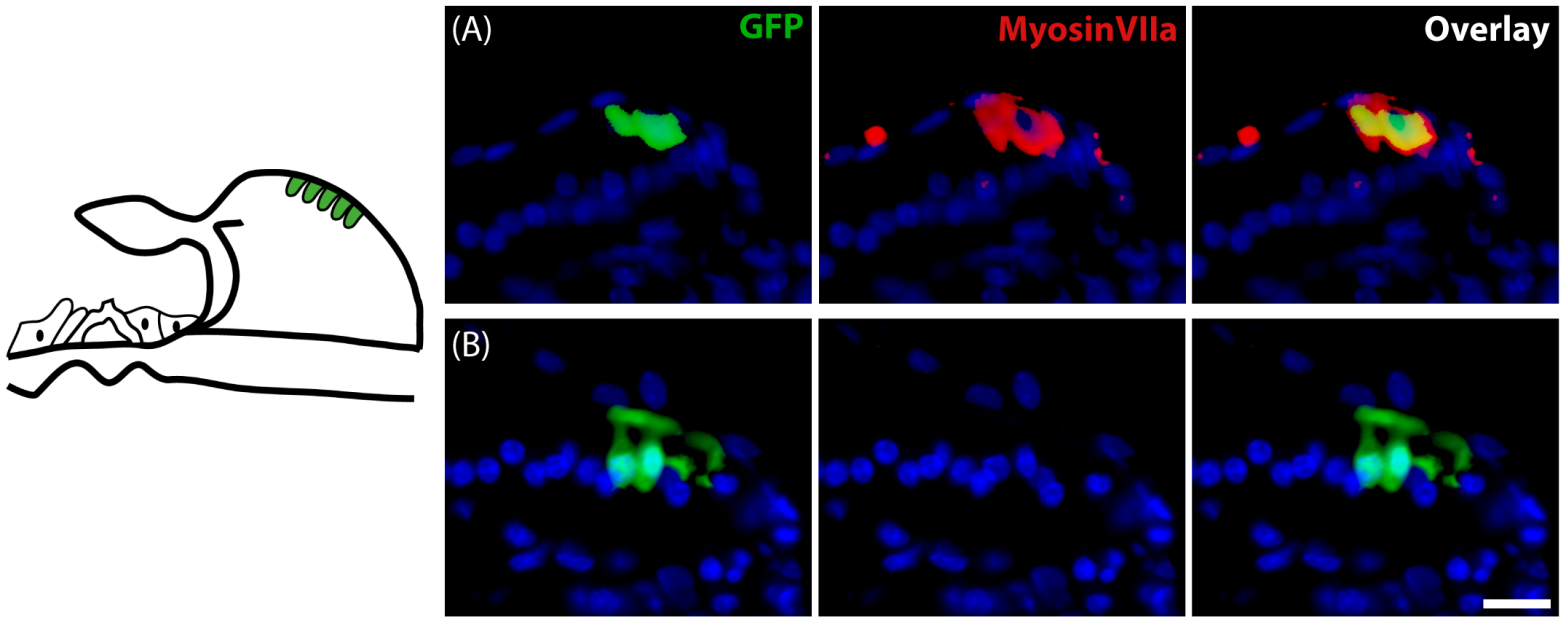
Transduction of interdental cells following ATOH1 or GFP treatment. Schematic at left illustrates the region of the interdental cells within the spiral limbus. The transduction of interdental cells (green) with ATOH1 (A) resulted in the expression of the HC marker myosinVIIa (red) in 85% of the cells. (B) MyosinVIIa expression was not detected following transduction of interdental cells with the GFP control vector. Scale bar  = 20 µm.

The interdental cells of the spiral limbus were often transduced and interestingly, when transduced by ATOH1, 85% of those GFP positive cells were also myosinVIIa-positive (Figure 3A). In only one instance was an interdental cell co-labelled with parvalbumin. When transduced with Ad-GFP only, no co-localisation with myosinVIIa or any other HC marker was observed in the interdental cells (Figure 3B).

#### Synaptic ribbon detection and quantification

**Figure 4:**
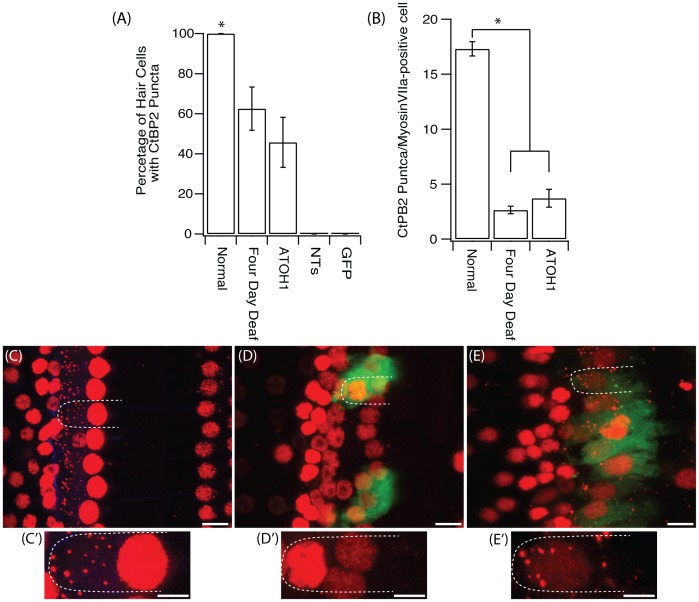
Fure 4. CtBP2 expression in IHCs. (A) There was a significant decrease in the percentage of HCs that were positive for CtBP2 puncta after four days of deafness and after treatment with ATOH1 when compared to the normal hearing cochlea (*p<0.05, one way ANOVA). (B) There were also significantly less CtBP2 puncta per IHC after four days of deafness or ATOH1 treatment. Examples of CtBP2 staining (red) in the (C) normal, (D) GFP treated and (E) ATOH1 treated cochlea with their respective magnified image (C′-E′), green  =  GFP. Scale bar  = 10 µm (main image) and 5 µm (magnified image)

A unique feature of IHCs in the cochlea is their ribbon synapse, which is critical for HC function. With the evidence that ATOH1 transduction induces cells to differentiate towards a HC phenotype, as noted by the expression of multiple HC markers, we next sought to determine if the transduced cells could also generate these highly specialised synaptic proteins. In normal cochleae all IHCs contained ribbon synapses, as indicated by the presence of CtBP2 puncta located on the basolateral surface of the HC (Figure 4A). When assessed four days post-deafening, of the IHCs that remained, only 62.5±10.7% (11 cells across 3 animals) had CtPB2 puncta. When examined three weeks post-ATOH1 gene therapy, 45.8±1.2% of myosinVIIa-positive cells were observed to have CtBP2 puncta. In total there were 21 myosinVIIa-positive cells with CtBP2-positive puncta in ATOH1 transduced cochleae across five animals (Figure 4A). In contrast, CtBP2 puncta were absent in the few remaining HCs observed after GFP or NTs treatment (Figure 4A). To further quantify the presence of synaptic ribbons in the normal hearing, four day deaf and ATOH1 treatment groups, the number of CtBP2 puncta per IHC was examined. In the cochleae of normal hearing animals there was a mean of 17.3±0.7 puncta/IHC, a value similar to that found in other species [14]. This number decreased 2.6±0.3 puncta/IHC in the four day deaf cohort and after ATOH1 treatment there were 3.7±0.8 puncta/IHC (Figure 4B). In the four day deaf animals and ATOH1 treated animals, the location of CtBP2 puncta also differed when compared to normal hearing animals. In the normal hearing cochlea, puncta were localised to the basolateral pole of the HC (Figure 4C). CtBP2 puncta were absent after treatment with GFP only (Figure 4D), whilst in the ATOH1-treated group puncta were observed in the middle and the apex as well as in the basolateral pole of the HC (Figure 4E).

### Peripheral fibre innervation of hair cells

**Figure 5:**
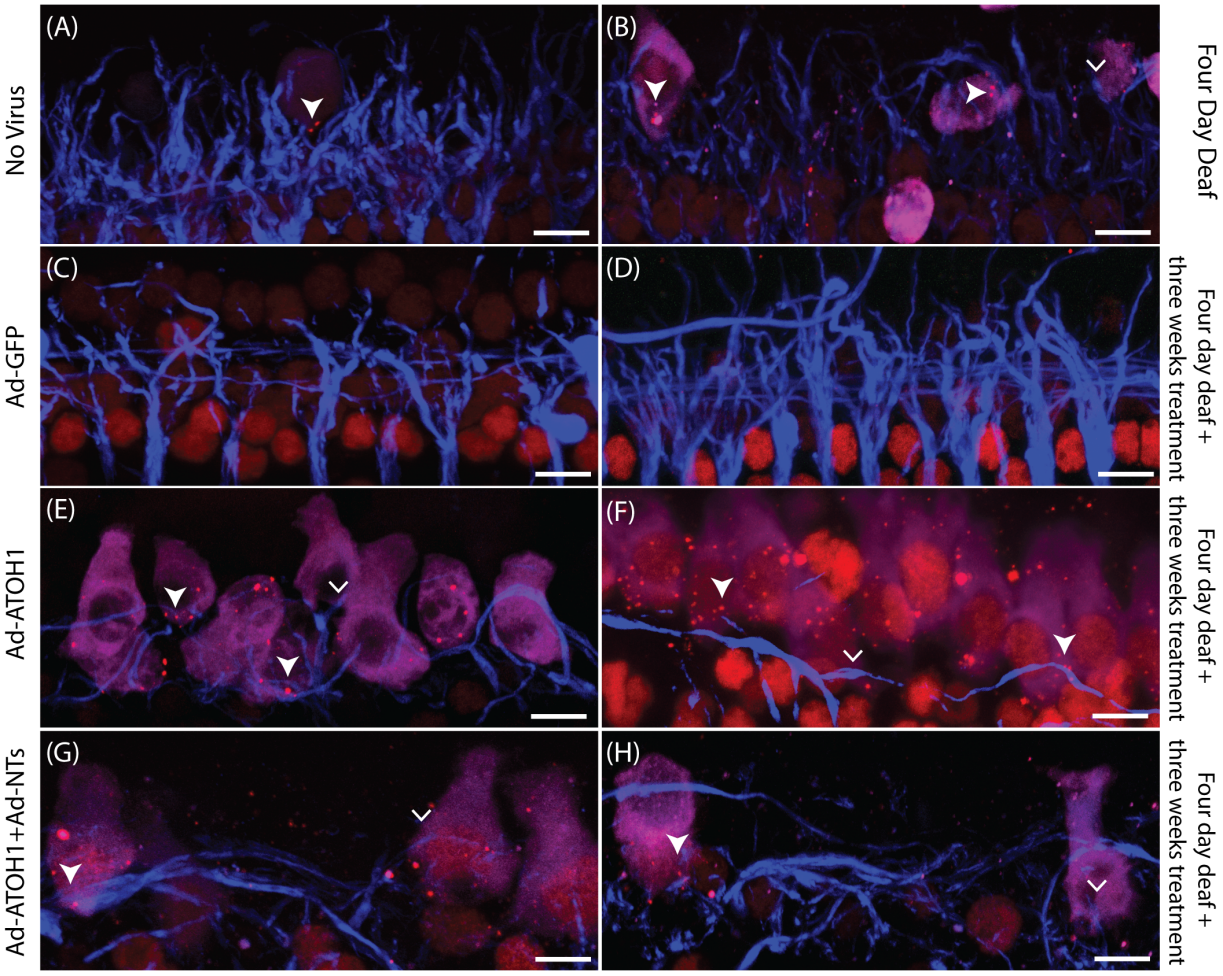
Surface preparations illustrating peripheral fibres in close proximity to HCs with ribbon synapses. Examples of peripheral fibres (blue) close to myosinVIIa-positive cells (magenta) with CtBP2 puncta (red; filled arrowhead) or without CtBP2 puncta (arrowhead) in the four day deafened (A&B), GFP treated (C&D), ATOH1 treated (E&F) or ATOH1+NTs (G&H) treated cochleae. Scale bar  = 10 µm.

Synapses were examined further to determine if peripheral auditory neuron fibres innervated myosinVIIa and CtBP2 puncta-positive cells. When examined four days post deafening there were very few remaining IHCs, and even fewer with CtBP2-positive puncta (Figure 5A, B). Of the HCs that were CtBP2-positive, there was a marked reduction in the density of peripheral fibres proximal to the remaining HCs, compared to the dense innervation pattern in the normal cochlea. Cochleae treated with Ad-GFP (Figure 5C, D) or Ad-NTs (not shown) did not have any CtBP2-positive puncta within transduced cells. In ATOH1 (Figure 5E, F) and the ATOH1+NTs (Figure 5G, H) cochleae, a number of peripheral fibres were observed to co-localise with HCs expressing CtBP2 puncta. There were also fibres observed within close proximity to myosinVIIa-positive cells without CtBP2 co-localisation. Interestingly, the peripheral fibres in the ATOH1 treated groups appeared to be travelling laterally along the myosinVIIa-positive cells, which is characteristic of OHC innervation rather than IHC innervation.

**Figure 6:**
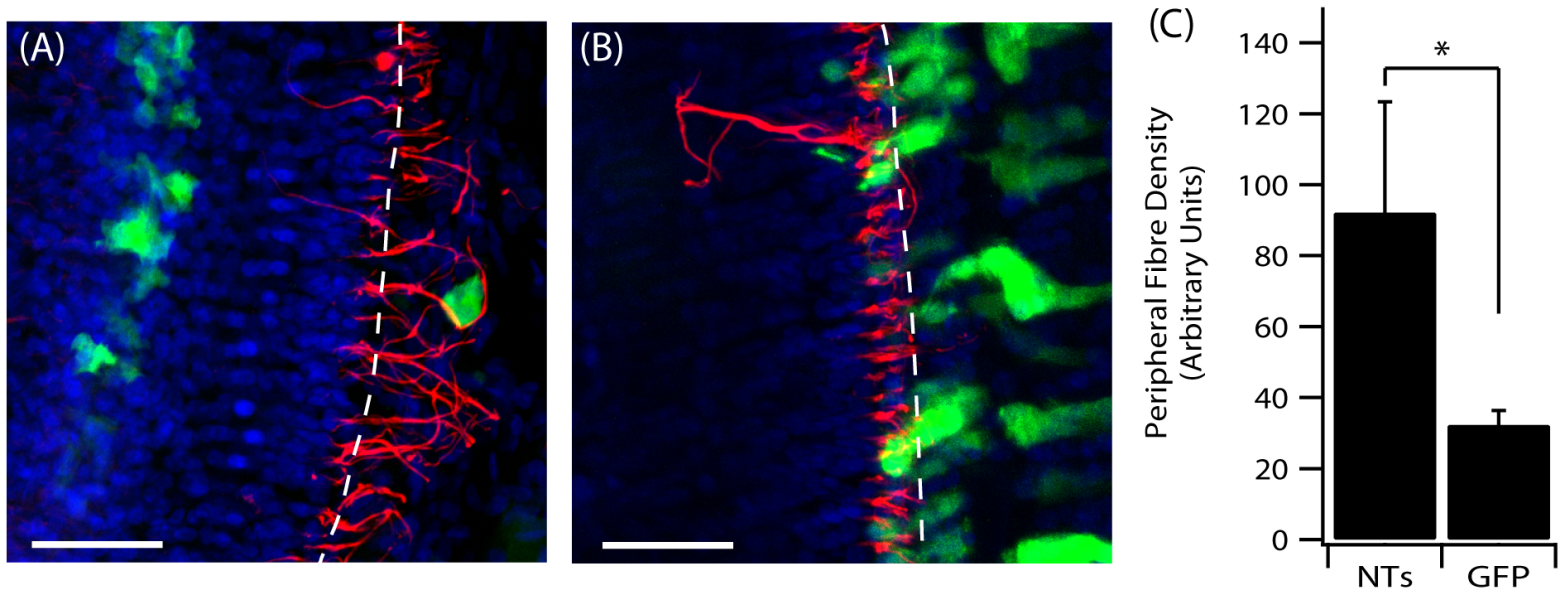
Resprouting peripheral fibres. Cochlear surface preparations were stained with anti-NF (red) and DAPI (blue). (A) Peripheral fibre responses to NT-expressing cells in the OC post Ad-NTs treatment. (B) Peripheral fibre response to GFP only expressing cells in the OC post Ad-GFP treatment. The perforated lines indicate the approximate medial edge of the inner pillar cells of the OC. Scale bar  = 50 µm. (C) Significantly greater fibre density was observed in close proximity (≤10 µm) to NT-secreting cells compared to GFP-only transduced cells (p<0.05, t-test).

Whilst treatment with Ad-NTs did not lead to an increase in the number of myosin-positive cells or CtBP2 puncta-positive cells, the density of peripheral fibres in close proximity to transduced cells was significantly greater in GPs treated with Ad-NTs (92.27±31.18 NF-pixels/GFP-positive cell) compared to GPs treated with Ad-GFP (32.34±4.04 NF pixels/GFP-positive cell; p<0.05, t-test). These peripheral fibres were observed to loop around the NT-expressing cells (Figure 6).

### Functional assessment of ATOH1 gene therapy

**Figure 7:**
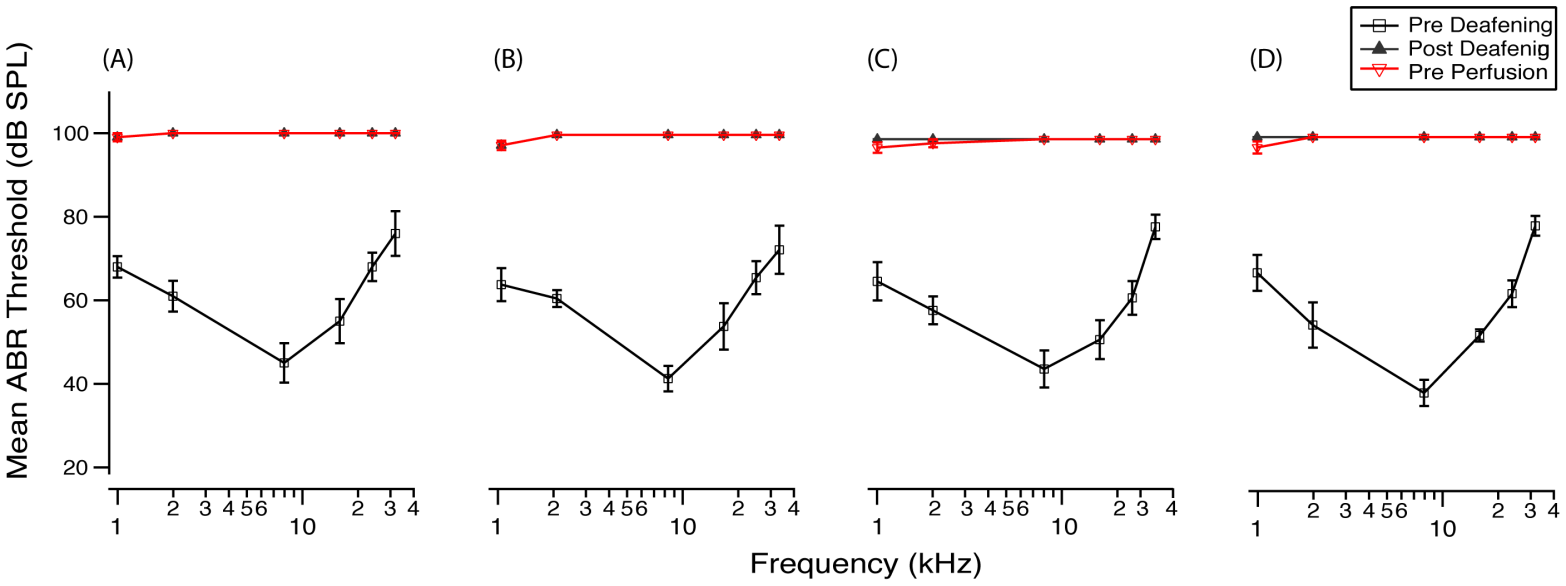
Mean ABR thresholds pre-deafening, post-deafening and post-treatment. There was a profound hearing loss across all frequencies measured (1, 2, 8, 16, 24, 32 kHz) four days post aminoglycoside deafening for all animals. Three weeks after treatment with either (A) Ad-GFP, (B) Ad-NTs, (C) Ad-ATOH1 or (D) Ad-ATOH1+Ad-NTs there was no change in hearing thresholds compared to those measured post-deafening (p>0.05, repeated measured ANOVA).

To test the functional effect resulting from the increase in HCs observed after ATOH1 gene therapy in the deaf cochlea, ABR thresholds were measured prior to aminoglycoside deafening, four days post deafening, and also three weeks post-gene therapy treatment (Figure 7). ABRs measured four days after deafening showed that all animals were profoundly deaf across all frequencies measured, indicating a loss of functional HCs (one way repeated measures ANOVA, p<0.001). When thresholds were remeasured three weeks post gene therapy treatment, there was no improvement in hearing thresholds in any of the treatment groups (one way repeated measures ANOVA, p>0.05). No differences were detected between hearing thresholds in the treated compared to the contralateral non-treated ear (data not shown), indicating no functional recovery due to ATOH1 or NT-gene therapy.

### Auditory neuron densities

To determine if the introduction of ATOH1 and the subsequent increase in HCs was able to promote the survival of auditory neurons after deafness, auditory neuron density was measured in the basal, middle and apical turns after Ad-injections. Statistical comparisons were made between injected and the non-injected contralateral cochlea for each cochlear region. There was no significant difference in auditory neuron density between the Ad-GFP treated cochleae and the contralateral cochleae (1101±29 versus 1125±53 auditory neurons/mm^2^), nor was there any difference in auditory neuron density between the treated and contralateral sides in the Ad-ATOH1 group (947±60 versus 933±57 auditory neurons/mm^2^). There was, however, a significant increase in auditory neuron density in the basal turn on the treated side compared to the contralateral side in the Ad-NTs group (1254±42 versus 1079±51 auditory neurons/mm^2^) and the Ad-ATOH1+NTs group (1168±84 versus 994±108 auditory neuron s/mm^2^; paired t-test, p<0.05).

## Discussion

The key finding of this study is that ATOH1-gene therapy results in an increase in the number of HCs, defined as myosinVIIa-positive cells with non-pyknotic nuclei residing within the OC, after profound aminoglycoside-induced SNHL in the mature GP. This study also demonstrated that cells transduced with ATOH1 expressed a multitude of HC proteins, however, these cells did not regenerate a full complement of synaptic ribbons nor was hearing function restored after severe SNHL.

### ATOH1 gene therapy was unable to restore hearing after deafness in the mature GP cochlea

Deafening resulted in a profound and rapid hearing loss across all frequencies, when measured four days post-deafening. In contrast to a previous study [10], the current study did not show any hearing recovery after the introduction of ATOH1. One possible explanation for this contrasting result is the differences in the severity of deafness and therefore the extent OC degeneration prior to ATOH1 introduction. The differing degree of severity of OC damage is illustrated by the contrasting ABR results. In a previous study there was a hearing response recorded in the contralateral ear when remeasured 8 weeks post-deafening [10], suggesting that there was a degree of recovery without treatment, unlike in the current study where complete hearing loss persisted in the non-treated contralateral ear when re-examined at all time points post-deafening. The level of recovery observed in this previous study, however, was greater in the ATOH1 treated ear suggesting, when viewed in the context of the current findings, that ATOH1 may have played a protective or repair role as well as a role in generating new HCs.

### ATOH1 gene therapy results in an increase in the number of HCs in the profoundly deaf cochlea

Whilst ATOH1 gene therapy was unable to restore hearing after aminoglycoside-induced SNHL, it did result in an increase in the number of HCs compared to cochleae examined four days post-deafening and compared to the contralateral non-treated cochleae. The number of HCs in the ATOH1 treated animal was, as expected, significantly less than that of the normal hearing animal. The numbers of HCs observed after ATOH1 therapy in this study, however, was less than that reported previously [10], where the number of IHCs observed in the inoculated ear was much closer to the number of IHCs observed in the normal hearing GP cochlea, again most likely reflecting the higher severity of damage to the OC in this study, limiting the number of protected or repaired HCs and the number of viable supporting cells that were able to undergo transdifferentiation after the deafening procedure used in this current study.

The HCs observed in the ATOH1 treated cochleae in the present study had a varied morphology; some displayed the more stereotypical mature pear-shaped IHC morphology, whilst others had a more spherical morphology (Figure 1). These observations are supported by an earlier study where ATOH1 treatment-induced HCs also lacked a mature morphology [13]. Given that there were occasional HCs present at the time of inoculation, it is possible that ATOH1 expression could be promoting the survival of existing HCs. As such, HCs with the more typical morphology might be existing HCs that were protected rather than regenerated HCs. Indeed, expression of ATOH1 within the supporting cells of the damaged OC was recently shown to repair stereocilia and improve hearing thresholds, suggesting that ATOH1 may be able to maintain and repair injured HCs [20]. However, the findings that the overall density of HCs in the ATOH1 inoculated cochleae were significantly greater than that observed in the four day deaf cohort (e.g. at the time point of ATOH1 inoculation), suggests that these new additional HCs were transdifferentiated from other cell types in the OC.

### ATOH1-transduced cells of the OC express multiple immature HC proteins

Upon further characterisation of the HCs observed after ATOH1 treatment, it was noted that the expression of HC markers was related to the location of the transduced cells, even within the OC region itself. Transduced cells in the inner sensory region, for example, expressed a multitude of HC markers (myosinVIIa, parvalbumin, calbindin), whilst expression of these HC markers by transduced cells was lower in the outer sensory region. It has been previously demonstrated that ectopic ATOH1 expression in the outer sensory region of the immature cochlea can lead to the generation of new HCs, which expressed myosinVIIa, parvalbumin and calbindin [13]. Interestingly, in both the current study and that conducted by Liu and colleagues (2012), cells that underwent transdifferentiation towards HC in the outer sensory region failed to express mature OHC proteins such as oncomodulin and prestin.

The varied expression observed within the OC region is thought to reflect both a location specific effect of ectopic ATOH1 expression within the OC region and the level of degeneration at the time of treatment, factors which have been previously shown to be a critical determinate in the ability of ATOH1 to manipulate non-sensory cells towards a HC fate [11], [21]. Specifically, the range of cells within the OC that are able to undergo ATOH1-mediated HC differentiation has been shown to decrease with age. By post-natal day eight in mice the subpopulation of supporting cells able to be driven towards a HC phenotype was restricted to the medial side of the IHC row. This location correlates to the location of the inner sensory region described in the present study. The lack of expression of HC markers by transduced endosteal cells and cells of the spiral ligament, most likely reflect an inherent inability of these cells to be transdifferentiated by ATOH1. Interestingly, the transduction of the interdental cells with Ad-ATOH1 resulted in expression of myosinVIIa, a marker not observed within these cells when transduced with the control (Ad-GFP) vector or in non-treated cochleae. The results presented demonstrate that ATOH1 is capable of forcing the transdifferentiation of some mature cochlear cells towards a HC phenotype. Not all HCs observed were the result of a protective effect afforded by ATOH1 treatment, as indicated by the greater number of HCs (as quantified by myosinVIIa positive cells in the sensory epithelium) after ATOH1 treatment, compared to the number of HCs present at the time of intervention. The direct transdifferentiation of non-sensory cells towards a HC phenotype through the introduction of ATOH1 has been further confirmed in another study, where approximately 97% of non-sensory cells that newly expressed myosinVIIa after the forced expression of ATOH1 were BrdU-negative, demonstrating that these myosinVIIa-positive cells did not undergo mitotic division prior to conversion towards a HC phenotype [9].

### ATOH1 gene therapy does not restore a full complement of synaptic ribbons in the deaf cochlea

The expression of the cohort of HC markers described herein demonstrates that the introduction of ATOH1 results in at least a partial change in phenotype towards a HC. The conversion of non-sensory cells to HCs has been observed in multiple studies [7], [10], [22], [23]. However, one requirement for mature functional connections is for HCs to have synaptic ribbons, which were assessed in this study by quantifying CtBP2-positive puncta staining. Of the remaining HCs observed after four days of hearing loss there was a significant decrease in the number of HCs that expressed CtBP2-positive puncta. Furthermore, the absolute number of puncta per HC was also significantly decreased at four days post deafening. A decrease in CtBP2-puncta positive HCs and in the number of synaptic ribbons per HC has been previously described after a reversible noise-induced hearing loss, suggesting that ribbon synapses are extremely sensitive to damage [24]. Treatment with ATOH1 was unable restore synaptic ribbons to normal levels, nor generate new HCs with synaptic ribbons at normal levels. Furthermore, the percentage of HCs that had CtBP2 puncta after ATOH1 treatment was not different to those examined after four days of deafness. Interestingly, no CtBP2 puncta were observed in HCs in the NTs or GFP treated cochleae. Collectively, these findings suggest that the ATOH1 may confer a level of protection on residual HCs and the delicate ribbon synapse as well forcing supporting cells to undergo transdifferentiation towards a HC phenotype.

While synaptic ribbons, albeit at a much lower number, were observed after ATOH1 treatment (alone and when combined with NTs) the regrowth of peripheral fibres towards the HCs and evidence of synaptogenesis between these HCs and auditory neurons still warranted investigation. We aimed to assess the connection between peripheral fibres and HCs, however, it was not possible to quantify synaptogenesis in this study due to the low number of remaining HCs that were positive for ribbon synapses combined with the low number of fibres observed. Unlike previous studies, which have qualitatively shown ectopic HC innervation [22], [23] and synaptic connections [8], there was no clear trend for the growth of peripheral fibres towards ectopic HCs nor towards HCs that where positive for CtBP2-positive puncta. Moreover, most fibres that were observed within close proximity of HCs appeared to travel laterally along the HCs, rather than form the synaptic boutons normally observed between auditory neuron afferent fibres and IHCs. In contrast, peripheral fibres in the Ad-NTs group did appear to respond to the gene therapy treatment. There were significantly more fibres within close proximity of the NT producing cell compared to GFP-only producing cells, indicating that NTs are able to influence the growth of peripheral fibres, similar to previous findings [15]. Interestingly, the combined treatment of ATOH1 and NTs did not result in an increase in HCs when compared to the four day deaf cohort, nor did this combined treatment appear to result in an increase in fibre resprouting. This effect is somewhat unexpected as the two vectors are unlikely to inhibit the effects on HC formation and fibre outgrowth observed when the vectors were injected alone. Therefore, the lack of HC regeneration and the lack of any apparent increase in fibre resprouting is likely to be due to the lower concentration of the individual viral titres, as when combined, the effective volume of each virus (Ad-ATOH1 and Ad-NTs) was halved.

## Conclusions

Collectively, the results presented in this study suggest that ATOH1-gene therapy may lead to an increase in HCs in the deaf cochlea in two ways; by promoting the survival and/or repair of the remaining HCs and by the direct transdifferentiation of supporting cells towards an immature HC phenotype. ATOH1 gene therapy alone, however, is unable to fully rescue the phenotype of surviving HCs. Nor is it able to cause the full conversion of non-sensory cells into mature HCs after severe aminoglycoside-induced deafness in the mature GP. The further examination of ATOH1 gene therapy should not be dismissed, as it still provides a powerful tool for the manipulation of non-sensory cells in the cochlea. When combined with other treatments such as manipulation of the Notch signalling pathway to decrease inhibitory signalling [25] or after forms of deafness that result in less severe damage to the OC, ATOH1 gene therapy may lead to functional improvement.

## Supporting Information

Data S1 **Data for supporting figures.** Hair cell counts: Number of hair cells in the inner sensory region of individual animals. This data was used to generate Figure 1. Hair cell expression: Number of GFP-positive cells co-expressing hair cell markers. This data was used to generate Figure 2. CtBP2 expression: Number of CtBP2 puncta per myosinVIIa-positive cell. This data was used to generate Figure 4. Peripheral fibres: Area of neurofilament-positive pixels within 10 mm of GFP-positive cells. This data was used to generate Figure 6. ABRs: Hearing thresholds of each animal pre-deafening, post-deafening and pre-perfusion. This data was used to generate Figure 7. SGN density: Density of spiral ganglion neurons in the basal, middle and apical turns of the cochleae of all animals. This data was used in the “Auditory Neuron Densities” section of the results.
(XLSX)
